# Lives Saved Through Increasing Adherence to Follow-Up After Abnormal Cervical Cancer Screening Results

**DOI:** 10.1097/og9.0000000000000001

**Published:** 2024-03-19

**Authors:** Diane M. Harper, Tiffany M. Yu, A. Mark Fendrick

**Affiliations:** Department of Obstetrics and Gynecology, the Department of Family Medicine, the Department of Bioengineering, School of Engineering, and the Center for Value-Based Design, University of Michigan, and the Department of Women’s and Gender Studies, University of Michigan College of Literature, Science and the Arts, Ann Arbor, Michigan; and Guidehouse, Inc, San Francisco, California.

## Abstract

**OBJECTIVE:**

To model the potential number of cancers prevented and life-years saved over a range of adherence rates to cervical cancer screening, surveillance follow-up, and follow-up colposcopy that may result from removing financial barriers to these essential clinical services.

**METHODS:**

A previously validated decision-analytic Markov microsimulation model was used to evaluate the increase in adherence to screening, surveillance, and colposcopy after an abnormal cervical cancer screening result. For each incremental increase in adherence, we modeled the number of cervical cancer cases avoided, the stages at which the cancers were detected, the number of cervical cancer deaths avoided, and the number of life-years gained.

**RESULTS:**

Compared with current adherence rates, the model estimated that an optimized scenario of perfect screening, surveillance, and colposcopy adherence per 100,000 women currently eligible for screening in the United States was 128 (95% CI, 66–199) fewer cervical cancers detected (23%), 62 (95% CI, 7–120) fewer cervical cancer deaths (20%), and 2,135 (95% CI, 1,363–3,057) more life-years saved. Sensitivity analysis revealed that any increase in adherence led to clinically meaningful health benefits.

**CONCLUSION:**

The consequences of not attending routine screening or follow-up after an abnormal cervical cancer screening result are associated with preventable cervical cancer morbidity and premature mortality. Given the potential for the removal of consumer cost sharing to increase the use of necessary follow-up after abnormal screening results and to ultimately reduce cervical cancer morbidity and mortality, public and private payers should remove cost barriers to these essential services.

## Abstract 2

Increased adherence to follow-up after an abnormal cervical cancer screening result will reduce the number of cancers and cancer deaths.

The clinical burden of cervical cancer in the United States has not changed much over 20 years^1^; although overall changes are minimal, racial and ethnic disparities continue to show increased incidence and mortality.^2^ Although all populations are eligible for screening, over the past decade, multiple reasons have driven the suboptimal uptake of cervical cancer screening, surveillance testing, and colposcopy. The Health Belief Model focuses on the fear of cancer, embarrassment from the speculum examination, lack of knowledge, anxiety, time away from work, transportation costs, childcare costs, and lack of insurance.^3^ Building on the lack of insurance, one potential and poorly studied factor is that many women with an abnormal initial cervical cancer screening test result (14–37%) do not receive clinically indicated follow-up diagnostic testing (20–75%) to determine the presence or absence of malignancy.

Cervical cancer screening itself—having received an A rating in 2018 from the U.S. Preventive Services Task Force—is among the preventive services covered without patient out-of-pocket costs (eg, copayments, deductibles) by the Patient Protection and Affordable Care Act of 2010.^4^ Despite the removal of financial barriers to initial cervical cancer screening (eg, cervical cytology, human papillomavirus [HPV] testing) for most insured women, the rate of screening for women 21–65 years of age has continued to decline, with inequities by race, education, income, and rurality.^5^ The current U.S. cervical cancer incidence^6^ (corrected for hysterectomy) has persisted at nearly three times the World Health Organization’s cervical cancer elimination rate goal.^7^

Although financial barriers for patients to initial cervical cancer screening have been eliminated for more than a decade, a 2022 claims analysis reported that 8 of 10 women undergoing a colposcopy incurred nontrivial out-of-pocket costs; those who required additional procedures (eg, colposcopy, endocervical curettage, endometrial biopsy; referred to hereafter as colposcopy+) faced up to hundreds of dollars more in costs. These out-of-pocket costs rose sharply during the 13 years studied. By 2019, a woman who received care beyond a biopsy could expect to face a total bill of nearly $1,000.

Given the robust evidence base demonstrating that a reduction in consumer cost sharing leads to modest increases in essential care, we hypothesized that with the removal of out-of-pocket costs for recommended follow-up diagnostic testing after an initial abnormal cervical cancer screening result, more women would initiate screening and use more surveillance testing and more colposcopy+ follow-up care would be delivered, ultimately leading to a reduction in care inequities, and fewer cases of cervical cancer in which the cervical cancer cases detected would be diagnosed at earlier and more treatable stages of disease. Accordingly, this study aimed to model the potential number of cancers prevented and life-years saved over a range of adherence rates to cervical cancer screening, surveillance testing, and follow-up diagnostic procedures that may result from removing financial barriers to these essential clinical services.

## METHODS

We evaluated the effect of increasing adherence to cervical cancer screening, surveillance testing, and follow-up colposcopy+ using a decision-analytic Markov microsimulation cost-effectiveness model; model design and inputs used have been described in detail.^8^ Briefly, a population of women aged 26–65 years with clinical and demographic characteristics of the prevalent population of women eligible for cervical cancer screening in the United States^9^ was entered into the model. Distributions by age (25–29, 30–39, 40–49, 50–59, 60–64 years), vaccination against high-risk HPV types 16 and 18, and probabilities of meeting cervical cancer screening exclusion criteria (human immunodeficiency virus [HIV], pregnancy, and hysterectomy) were extracted from data published by the U.S. Census Bureau, Agency for Healthcare Research and Quality, and Centers for Disease Control and Prevention.^10–15^ Clinical characteristics, including high-risk HPV status by age (negative, 16/18–positive, 12 other–positive), and histology status of cervical tissues (normal, cervical intraepithelial neoplasia [CIN] 1, CIN 2, CIN 3, invasive cervical cancer stage 1) by high-risk HPV genotype were informed by published studies.^8,16^

This analysis simulated 98,000 women, evenly divided across patient groups defined by age, high-risk HPV status, and histology status expected to be present in the U.S. population (49 patient groups, 2,000 patients per group). Each patient was simulated on an annual cycle under the current and improved adherence paradigms from model entry to death. Patient microsimulations were then aggregated by patient group, and results were averaged across groups and weighted by the prevalence of each group in the United States to address equity in population distribution. We used 95% CIs by 1,000 bootstrap samples of 98,000 microsimulated patients to assess uncertainty.

Because most cervical cancer screening in the United States is high-risk HPV and cervical cytology cotesting, we applied this screening strategy as the baseline in the model. Half of the simulated patients used pooled high-risk HPV testing, and the other half used high-risk HPV genotyping.^17^ We used risk-based management strategies per the 2019 ASCCP guidelines.^18^ Three-year surveillance intervals were applied for patients with abnormal results whose risk threshold for CIN 3 or higher was between 0.15% and 0.54%. One-year surveillance intervals were modeled for patients with abnormal screening results whose risk threshold for CIN 3 or higher was 0.55–4%. Patients with abnormal results whose risk threshold for CIN 3 or higher was above 4% received immediate colposcopy and additional procedures as clinically indicated.

Those undergoing colposcopy+ and found to have CIN 2 or CIN 3 were recommended a loop electrosurgical excision procedure (LEEP) treatment (adherence rate 60%). Patients for whom the ASCCP guidelines allowed either colposcopy or treatment (those whose initial abnormal screening results were associated with a risk of CIN 3 or higher between 25% and 59%) were assigned to immediate treatment with LEEP. Patients needing colposcopy or LEEP were modeled to return for a surveillance visit the following year.

Patients at age 65 no longer received cervical cancer screening in the model if they 1) had completed their most recent screening, 2) did not have abnormal screening test results within the past decade, and 3) did not undergo LEEP over the previous 25 years.

The literature informed adherence rates with screening and follow-up recommendations (eg, returning for subsequent surveillance visits, undergoing colposcopy+ or LEEP) under the current algorithm. The initial seeding of the model is presented in Table 1. The algorithm is presented in Appendix 1, available online at http://links.lww.com/AOG/D606. Patients receiving prior normal results had a 70% probability of returning for screening after a 5-year interval; that is, there was a 70% chance that the patient would return for routine screening in 5 years. If the patient did not return in the recommended year, there was a 70% chance applied in each subsequent year until the patient did return.

**Table 1.** Input Values for Adherence With ASCCP Guidelines–Recommended Cervical Cancer Screening

Input	Modeled Paradigm (%)	
	Current Practice	Improved Adherence
Probability of attending a recommended screening visit		
Without abnormal results at the prior screening^5^,*	70	100
After abnormal results at the prior screening^19^,†	40	100
Probability of complying with recommended procedures		
Colposcopy^19^	70	100
LEEP^20^	60	
Probability of going straight to procedure when returning after nonadherence‡		
Colposcopy	50	
LEEP^21^	50	

LEEP, loop electrosurgical excision procedure.

* After a prior screening for which ASCCP guidelines recommended a 5-year screening interval. This is also applied at model entry.

† After a prior screening for which ASCCP guidelines recommended a 3-year screening interval, 1-year screening interval, colposcopy, or treatment.

‡ Assumption: Applicable if the woman returns for a procedure in the year immediately after initial nonadherence. If returning in later years, the woman is modeled to start again with testing.

Similarly, patients who needed a 1- or 3-year surveillance interval or a 1-year return after undergoing colposcopy or LEEP were considered to be returning after prior abnormal results and had a 40% probability of adherence. If the patient did not return in the recommended year, a 40% chance was applied in each subsequent year until this visit occurred.

We modeled adherence to colposcopy at 70% in the year of recommendation, according to on published findings from an analysis of three large-scale surveys of women in the United States (Behavioral Risk Factor Surveillance System, the Health Information National Trends Survey, and the Health Center Patient Survey).^5^ Women who undergo colposcopy may also receive endocervical biopsy if clinically indicated.^22^ Adherence to LEEP was modeled at 60% in the year of recommendation on the basis of published results from the New Mexico HPV Pap Registry.^20^ Women who did not receive colposcopy or LEEP as recommended within the year were estimated to return in subsequent years, with the probability of adherence based on the previously missed procedure applied each year. If the patient returned in the year immediately following, the model applied a 50% probability that the patient can go directly to the previously missed procedure (ie, to colposcopy or LEEP). Otherwise, or if the patient did not return until later years, the patient was assumed to undergo cotesting surveillance at the return visit.

The model tested the effect of increasing the probability of adherence to returning for subsequent routine screenings, surveillance follow-up, and colposcopy+ over a range from baseline to perfect adherence (100%) to illustrate the potential health benefits of enhanced uptake. The adherence to LEEP was held constant.

The modeling of cervical cancer prevention, diagnosis, and treatment and the effect of the natural history of the disease have been robustly described.^8^ In brief, inputs for the performance of high-risk HPV tests, cervical cytology tests, and colposcopy and biopsy were derived from published validation trials and studies of cervical cancer screening.^16,22–24^ Published studies and meta-analyses of the effect of surveillance, HPV vaccination,^25–33^ and progression with undiagnosed invasive cervical cancer informed^34^ the annual health state transition probabilities for high-risk HPV infection, clearance, and development or regression of CIN 1–3. The diagnosis of invasive cervical cancer could be made 1) by way of patient symptoms,^34^ 2) through colposcopy with biopsy, or 3) in the year before cancer death. Cancer-specific and other causes of mortality rates were determined from Centers for Disease Control and Prevention data and the National Cancer Institute's Surveillance, Epidemiology, and End Results U.S. cancer registry database.^35,36^ Increased cancer-specific mortality risk in the absence of invasive cervical cancer diagnosis used comparisons of real-world data from patients who did compared with those who did not receive treatment in the other cancer registry databases.^37^

The study was deemed exempt by the University of Michigan IRBMED because no individual patient information was used.

## RESULTS

**Fig. 1.**
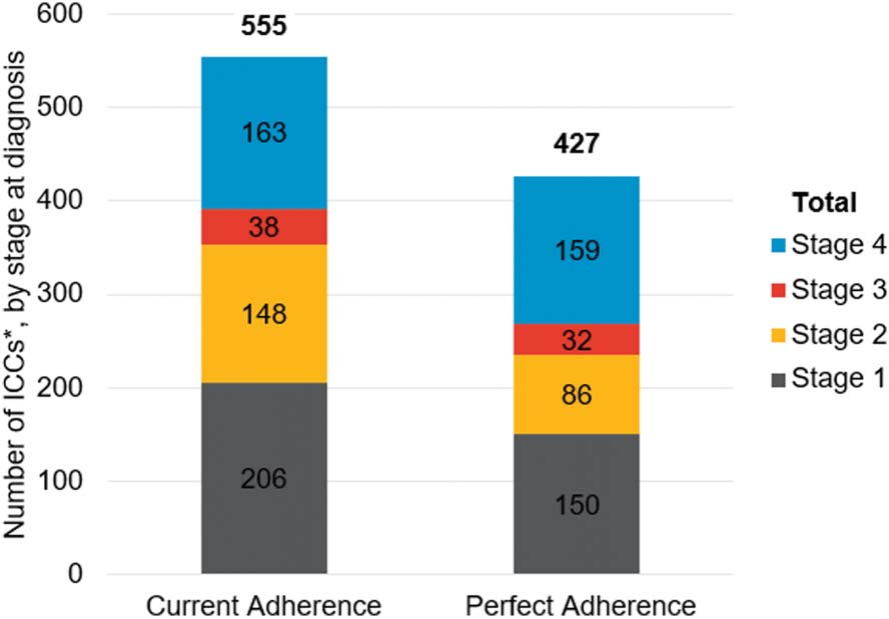
Detected cervical cancers by stage at diagnosis, with current vs perfect adherence to screening and surveillance and colposcopy with biopsy, endocervical curettage, or endometrial biopsy. Because of increased adherence to screening and surveillance and colposcopy+ follow-up, there were 128 fewer cancers/100,000 women currently eligible for screening. A total of 92% of the cancers were detected at the earliest stages, when there is a greater chance for cure with treatment. *Per 100,000 women currently eligible for screening over their lifetimes. ICC, invasive cervical cancer.

Our results show a reduction in cervical cancers and a reduction in associated deaths with increasing uptake of screening-related services. When adherence to cervical cancer screening, surveillance, and colposcopy+ examinations was projected to be 100% for women currently eligible for cervical cancer screening (ie, best case scenario), the simulation estimated that 128 (23%) fewer cervical cancers resulted per 100,000 women eligible for screening over a lifetime (95% CI, 66–199); that is, perfect adherence leads to 427 cervical cancers detected per 100,000 women currently eligible for screening over a lifetime, whereas current adherence has 555 cancers developing per 100,000 women currently eligible for screening over a lifetime (Table 2). The stage distribution of the 128 fewer cervical cancer cases that developed under the perfect adherence scenario was detected predominantly in the earlier stages (Fig. 1).

**Table 2.** Potential Lifetime Benefit With Perfect Screening and Surveillance and Colposcopy+ Adherence (95% CI) Per 100,000 U.S. Women in the Current Screening-Eligible Population

Cancers Avoided	Cancer Deaths Avoided	Life-Years Saved
−128 (−199 to −66)	−62 (−120 to −7)	2,135 (1,363 to 3,057)

*Harper. Improving Adherence after Abnormal Cervical Cancer Screening. O&G Open 2024*.

When the perfect screening, surveillance, and colposcopy+ adherence was entered into the model, it estimated that 62 (95% CI, 7–120) cervical cancer deaths per 100,000 women currently eligible for screening were prevented (305/100,000 [current] vs 243/100,000 [perfect adherence], a 20% decrease in cancer deaths). This mortality reduction translated into 2,135 (95% CI, 1,363–3,057) life-years saved per 100,000 women currently eligible for screening.

Table 3 represents a two-way sensitivity analysis to examine various adherence rates for screening and surveillance compared with colposcopy+. At the current colposcopy+ attendance rate of 70% and as adherence to screening and surveillance increases from 70% and 40% to 100% and 100%, the number of life-years gained maximized at 1,840 per 100,000 women currently eligible for screening. The model estimated that when incremental increases in adherence to screening and surveillance were achieved, with no change from the current colposcopy+ adherence rate, substantial life-years were still gained. For example, when the screening and surveillance was increased to 90% and 80% and the colposcopy+ adherence rate stayed at the current 70%, the life-years gained would be 880 per 100,000 women currently eligible for screening.

**Table 3.** Two-Way Sensitivity Analysis: Potential Life-Years Gained With Improved Adherence in Screening and Surveillance and Colposcopy+ After an Abnormal Cervical Cancer Screening Result*

Adherence to Screening (%)		Adherence to Colposcopy (n)			
Routine	Surveillance†	70%	80%	90%	100%
70	40	0	84	328	359
80	60	713	530	629	735
90	80	880	1,076	1,167	1,245
100	100	1,840	1,996	2,045	2,135

* The base case modeled the current adherence to screening, returning for surveillance (follow-up after an abnormal screening result below the colposcopy+ threshold), returning for colposcopy+ (after a higher risk abnormal screening result), and returning for surgical excision after a cervical intraepithelial neoplasia 2 or 3 diagnosis and is the referent at zero life-years/100,000 patients eligible for screening gained.

† Defined as visits on an ASCCP 2019 guideline–recommended 1- or 3-year follow-up interval.

Using current screening and surveillance rates as the base case rate, the model estimated that increasing the adherence rate of colposcopy+ from 70% to 100% would add up to 359 additional life-years per 100,000 women currently eligible for screening. The two-way sensitivity analysis (Table 3) demonstrates that, when baseline screening and surveillance adherence is increased to 80% and 60% and colposcopy+ adherence improved to 80%, 530 per 100,000 life-years were gained. At an 80% and 60% rate for screening and surveillance, the perfect uptake of colposcopy+ leads to 735 per 100,000 life-years gained, nearly 40% more life-years gained (205/100,000 women currently eligible for screening).

The current U.S. cervical cancer incidence is 11.5 per 100,000.^38^ Our model population reflected a cross-section of the women currently eligible for cervical cancer screening in the United States at model entry and followed them to death, resulting in an average time in model of 38 years and 128 cervical cancers averted per 100,000 women currently eligible for screening. This finding can roughly translate to a 30% annual reduction (3.4 fewer cases/100,000). Similarly, using the current U.S. cervical cancer mortality rate (3.0/100,000),^38^ the model estimated that perfect adherence to the cervical cancer screening continuum would reduce the annual mortality rate by half (1.6/100,000 reduction in the annual mortality rate).

## DISCUSSION

The clinical burden of cervical cancer has not changed over 20 years,^1^ yet cervical cancer–related expenditures have increased substantially. The total U.S. annual medical cost of cervical cancer care in 2020 was $2.3 billion,^39^ driven mainly by cervical cancer cases detected at later stages that are most expensive to treat. Our model showed that increasing adherence to screening and surveillance and colposcopy+ could reduce the number of cancers and shift those detected to earlier stages, allowing more effective and less expensive treatment with the potential to reduce cancer-related mortality by half in the best-case scenario.

Current cervical cancer incidence per 100,000^38^ is significantly higher for Black (16.8), Hispanic (15.8), and American Indian/Native American (11.9) women than non-Hispanic White women (10.0). Likewise, mortality per 100,000 women is significantly worse among Black (5.0), Hispanic (3.5), and American Indian/Native American (4.0) women than non-Hispanic White women (2.6). The results of the model suggest that substantial improvements in preventing cancers and avoidable deaths can be achieved by increasing adherence to screening, surveillance, and colposcopy+. The racial inequities are more likely to be affected by the removal of out-of-pocket costs of surveillance follow-up and diagnostic colposcopy+ examinations^40^ because Black and Hispanic women and those with lower incomes disproportionately forgo these essential clinical services as a result of cost.^39^

Lessons from recent policies that addressed out-of-pocket costs for follow-up care after an initial screening test recently implemented for colorectal cancer screening apply to cervical cancer screening. The 2022 “FAQs About Affordable Care Act Implementation Part 51” by the Biden administration required that commercial insurers eliminate cost sharing for diagnostic colonoscopy after an abnormal noninvasive colorectal cancer screening test result.^41^ A similar policy was adopted by the Medicare program soon thereafter. A recent publication modeling the potential clinical and economic effects of this policy of removing out-of-pocket costs for follow-up colonoscopy estimated up to a 26% increase in the number of life-years gained and net cost savings with a modest follow-up care increase.^42,43^

Prior work shows that out-of-pocket costs for follow-up to colposcopy+ were frequent and nontrivial and increased significantly over time.^44^ It is well known that removing cost sharing boosts the use of preventive services, which helps to decrease disparities and save lives.^45^ The 2018 U.S. Preventive Services Task Force guidelines for cervical cancer prevention include the Grade A recommendation for an HPV-based screening program with or without cytology, allowing all screening to be covered without out-of-pocket costs for all recommended ages. The U.S. Preventive Services Task Force guidelines also state, “Strategies that aim to ensure that all women are appropriately screened and receive adequate follow-up are most likely to succeed in further reducing cervical cancer incidence and mortality in the United States.” The findings of this simulation provide a compelling argument that 1) many lives could be saved if more women undergo appropriate follow-up examinations after screening, 2) cervical cancer inequities could be mitigated, and 3) associated reductions in cervical cancer could lead to the United States reaching the World Health Organization’s elimination rate goal.

Our model is the first evaluation of increasing adherence from current practice to perfect follow-up after abnormal cervical cancer screening results in the literature, in which we accounted for HPV vaccination status, age of screening, and population weights to match the U.S. population. We have used the best available literature data for natural history and clinical disease development. However, actual clinical outcomes may differ from modeled results.

The Preventive Services Provision of the Patient Protection and Affordable Care Act mandates the coverage of initial cervical cancer screening without cost sharing. However, this policy does not require payers to cover the entire cancer screening continuum without cost sharing. The failure to follow up on a positive screening test in a manner that is concordant with evidence-based guidelines undermines the screening process and can delay diagnosis. Payers should follow the American Cancer Society position statement that declares that cancer screening should be understood as a continuum of testing rather than a single screening test and advocates for the elimination of out-of-pocket costs for a full continuum of cancer prevention tests for all screening-detectable cancers as integral and necessary to resolve whether an adult undergoing screening has cancer.^46^

The consequences of not attending routine screening or testing after an abnormal cervical cancer screening results are associated with preventable cervical cancer morbidity and premature mortality.^47,48^ It is well established that small out-of-pocket costs are a barrier to receiving health care.^49^ Removing cost barriers increases the uptake of preventive care in all populations, especially in populations with health inequities.^45^ The consequences of not attending routine screening or testing after an abnormal screening result are associated with cancer progression and premature mortality.^47,48^ Finally, the costs of the surveillance and diagnostic follow-up tests continue to increase, making adherence to follow-up even less affordable, leading to financial toxicity.^50^

Our modeling estimates the potential substantial health benefits that could result in incremental adherence improvements to follow-up after abnormal cervical cancer screening results. To better achieve the full benefit of cervical cancer screening, public and private payers must cover the full screening continuum without cost sharing for these recommended surveillance and colposcopy+ examinations, regardless of the patient's designated risk. This would be consistent with the 2022 frequently asked questions specifying no patient cost sharing for follow-up colonoscopy after a positive noncolonoscopy colorectal cancer screening examination result.
